# Sphingosine-1-phosphate receptor inhibition prevents denervation-induced dendritic atrophy

**DOI:** 10.1186/s40478-016-0303-x

**Published:** 2016-03-31

**Authors:** Laurent M. Willems, Nadine Zahn, Nerea Ferreirós, Klaus Scholich, Nicola Maggio, Thomas Deller, Andreas Vlachos

**Affiliations:** Institute of Clinical Neuroanatomy, Neuroscience Center, Goethe-University Frankfurt, Theodor-Stern-Kai 7, Frankfurt, 60590 Germany; Institute of Clinical Pharmacology, Pharmazentrum Frankfurt, ZAFES, Goethe-University Frankfurt, Frankfurt, 60590 Germany; Talpiot Medical Leadership Program, Department of Neurology, The Chaim Sheba Medical Center, Tel HaShomer, 52621 Israel; Sackler Faculty of Medicine and Sagol School of Neuroscience, Tel Aviv University, Tel Aviv, 6997801 Israel; Present Address: Institute for Anatomy II, Faculty of Medicine, Heinrich-Heine-University, Duesseldorf, 40225 Germany

**Keywords:** Entorhinal cortex lesion, Brain injury, Multiple sclerosis, Lipid signaling, Structural plasticity, Neuroinflammation

## Abstract

A hallmark of several major neurological diseases is neuronal cell death. In addition to this primary pathology, secondary injury is seen in connected brain regions in which neurons not directly affected by the disease are denervated. These transneuronal effects on the network contribute considerably to the clinical symptoms. Since denervated neurons are viable, they are attractive targets for intervention. Therefore, we studied the role of Sphingosine-1-phosphate (S1P)-receptor signaling, the target of Fingolimod (FTY720), in denervation-induced dendritic atrophy. The entorhinal denervation in vitro model was used to assess dendritic changes of denervated mouse dentate granule cells. Live-cell microscopy of GFP-expressing granule cells in organotypic entorhino-hippocampal slice cultures was employed to follow individual dendritic segments for up to 6 weeks after deafferentation. A set of slice cultures was treated with FTY720 or the S1P-receptor (S1PR) antagonist VPC23019. Lesion-induced changes in S1P (mass spectrometry) and S1PR-mRNA levels (laser microdissection and qPCR) were determined. Denervation caused profound changes in dendritic stability. Dendritic elongation and retraction events were markedly increased, resulting in a net reduction of total dendritic length (TDL) during the first 2 weeks after denervation, followed by a gradual recovery in TDL. These changes were accompanied by an increase in S1P and S1PR1- and S1PR3-mRNA levels, and were not observed in slice cultures treated with FTY720 or VPC23019. We conclude that inhibition of S1PR signaling prevents dendritic destabilization and denervation-induced dendrite loss. These results suggest a novel neuroprotective effect for pharmaceuticals targeting neural S1PR pathways.

## Introduction

During the past years considerable effort has been made to better understand the pathophysiological mechanisms underlying neuronal cell loss or alterations in synaptic transmission in neurological and psychiatric diseases [1, 2]. However, far less attention has been dedicated to secondary injuries, e.g., neuronal denervation and atrophy, which invariably occur after traumatic, ischemic, haemorrhagic, neurodegenerative or neuroinflammatory brain damage in areas connected to the primary lesion site [3, 4]. These transneuronal effects on the neuronal network contribute considerably to the clinical symptoms [5, 6]. Moreover, maladaptive structural and functional changes in deafferented, but otherwise healthy brain regions have been implicated in several lesion-related long-term complications such as pain, epilepsy or memory dysfunction [7].

The role of *structural and functional disconnection* is of particular interest in Multiple Sclerosis (MS), since some of the cognitive deficits and other symptoms seen in patients have been attributed to perturbations of network function [5, 6]. Current pharmacological treatment focuses on the primary mechanism of injury and aims at modulating the immune system in order to prevent axonal damage and cell loss [8–10]. Secondary changes, triggered by denervation-induced transneuronal alterations, have so far not been considered a target. Interestingly, recent experimental evidence suggests that several immune mediators and inflammatory signaling pathways influence neuronal plasticity [1, 11–13]. Among them are sphingosine-1-phosphate (S1P) and its signaling pathways [14, 15], which are the targets of the oral immune-modulating drug Fingolimod (FTY720), now widely used in MS-therapy [16–18]. Therefore, we hypothesized that S1P-receptor (S1PR) modulation interferes with secondary brain injury by acting directly on neural tissue.

**Fig. 1:**
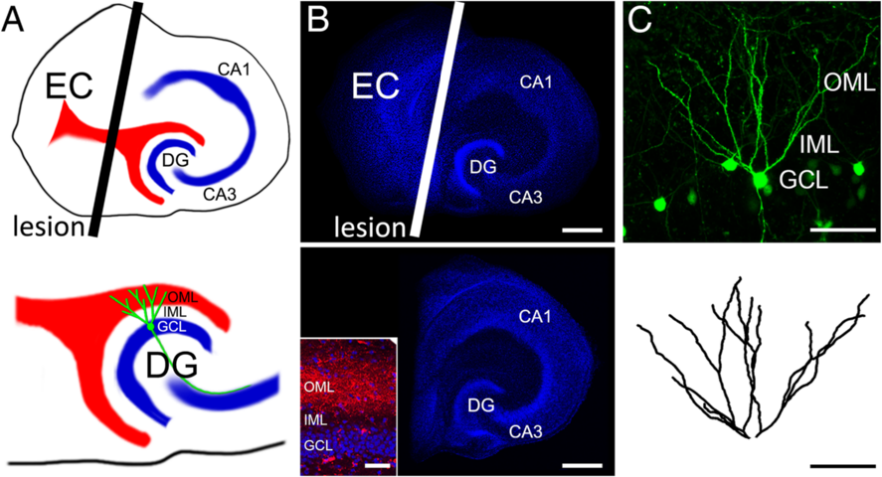
Entorhinal denervation in vitro model. **a** Schematic of an organotypic entorhino-hippocampal slice culture. The entorhino-hippocampal projection (red), which originates in the entorhinal cortex (EC) and terminates in the outer molecular layer (OML) of the dentate gyrus (DG) is transected with a sterile scalpel (black line; plane of transection, top). This lesion leads to a partial denervation of dentate granule cells (green schematic cell shown in the magnification of the DG, bottom) without directly damaging the target region (CA1, hippocampal subfield Cornu Ammonis 1; CA3, hippocampal subfield CA3; GCL, granule cell layer; IML, inner molecular layer; OML, outer molecular layer). **b** A non-denervated (top) and denervated (bottom) three-week old slice culture stained with TO-PRO (blue, nuclear stain). To assure a complete and reproducible denervation of the DG in all experiments, the EC was removed from the culturing dish. The inset shows Mini-Rubi traced (red) entorhinal fibers terminating in the OML of the DG. Scale bar: 200 μm (inset: 50 μm). **c** Entorhino-hippocampal slice cultures prepared from Thy1-GFP mice were employed to visualise individual dentate granule cells of denervated cultures and age- and time-matched non-denervated controls using time-lapse microscopy. An example of a GFP-expressing granule cell is shown (2D-projected confocal image stack). Dendritic trees of dentate granule cells were manually reconstructed in 3D-confocal image stacks. Scale bars: 100 μm

To address this hypothesis, we used an established in vitro denervation model (Fig. 1; [19, 20]) and studied the role of S1PR signaling in the prevention of denervation or disconnection damage. Time-lapse microscopy was used to assess the dynamics of denervated neurons under control conditions and following axonal denervation over a period of up to 6 weeks [21, 22]. Our results demonstrate that S1P signaling is involved in the remodeling of denervated brain regions and propose that drugs interfering with S1PRs, i.e., FTY720, prevent the denervation-induced loss of dendrites. These findings provide further evidence for a direct action of FTY720 on neural tissue. Furthermore, our results suggest that drugs targeting S1PR signaling could prove to be of value as disease-modifying drugs in several major neurological diseases, since this pharmacologic approach appears to target a widespread and important secondary disease mechanism, which is independent of the mechanisms leading to neuronal cell death at the primary lesion site.

## Materials and methods

### Preparation and maintenance of slice cultures

Experimental procedures were performed in agreement with the German law on the use of laboratory animals and approved by the animal welfare officer of Goethe-University (Faculty of Medicine). Entorhino-hippocampal slice cultures were prepared at postnatal day 4–5 from Thy1-GFP mice [23] of either sex as previously described [22, 24]. In these cultures a subset of neurons expresses GFP, which allows for the visualization of neurons in living tissue (Fig. 1). Cultivation medium contained 50 % MEM (v/v), 25 % basal medium eagle (v/v), 25 % heat-inactivated normal horse serum (v/v), 25 mM HEPES buffer solution, 0.15 % bicarbonate (w/v), 0.65 % glucose (w/v), 0.1 mg/ml streptomycin, 100 U/ml penicillin, and 2 mM glutamax. pH was adjusted to 7.3 and medium was replaced three times per week. All slice cultures were allowed to mature for 18–20 d in a humidified atmosphere with 5 % CO_2_ at 35 °C before they were used for experiments.

### Entorhinal denervation

Slice cultures show an organotypic morphology [24]. In slice cultures of entorhinal cortex and hippocampus the entorhino-dentate fiber tract, i.e., perforant pathway is present (Fig. 1a, b) and terminates on dentate granule cells in an organotypic pattern. In mature (18–20 days in vitro) mouse slice cultures this innervation pattern is stable and can be studied for several weeks in vitro using time-lapse imaging [21, 22]. Using a sterile scalpel blade we transected the entorhino-dentate fiber tract under visual control by cutting through the culture from the rhinal fissure to the hippocampal fissure. To ensure complete and permanent separation of the entorhinal cortex from the hippocampus, the entorhinal cortex was subsequently removed in every denervation experiment and only the de-entorhinated hippocampus remained in the dish (Fig. 1b). Of note, in previous studies we have shown that this procedure does not directly damage the target neurons in the dentate gyrus. Rather, this mechanical transection results in a highly standardized and reproducible loss of entorhinal axons in the outer molecular layer (see inset in Fig. 1b). The distal dendrites of dentate granule cells (Fig. 1c, d) are heavily denervated and lose a considerable portion of their synaptic inputs (~85–90 % of synapses in vivo [25]).

### Perforant path-tracing

For anterograde tracing of the entorhino-hippocampal pathway a biotinylated and rhodamine-conjugated dextranamine (“mini-rubi”, Molecular Probes, Life Technologies, USA) crystal was placed on the entorhinal cortex [26, 27]. 3–4 days later cultures were fixed in a solution of 4 % (w/v) paraformaldehyde and 4 % (w/v) sucrose in phosphate buffered saline (PBS) for 1 h, then washed thoroughly and coverslipped with fluorescent mounting medium (DAKO, Germany). For nuclear staining cultures were incubated with Topro-3-iodide (1:5000 in PBS for 10 min; Invitrogen, USA). Traced entorhino-hippocampal fibers were visualized using a Nikon Eclipse C1si laser-scanning microscope equipped with a 40× oil-immersion (NA 1.3, Nikon) and 60× oil-immersion (NA 1.4, Nikon) objective lens (Fig. 1b).

### Long-term time-lapse imaging of dentate granule cells in slice cultures

**Fig. 2:**
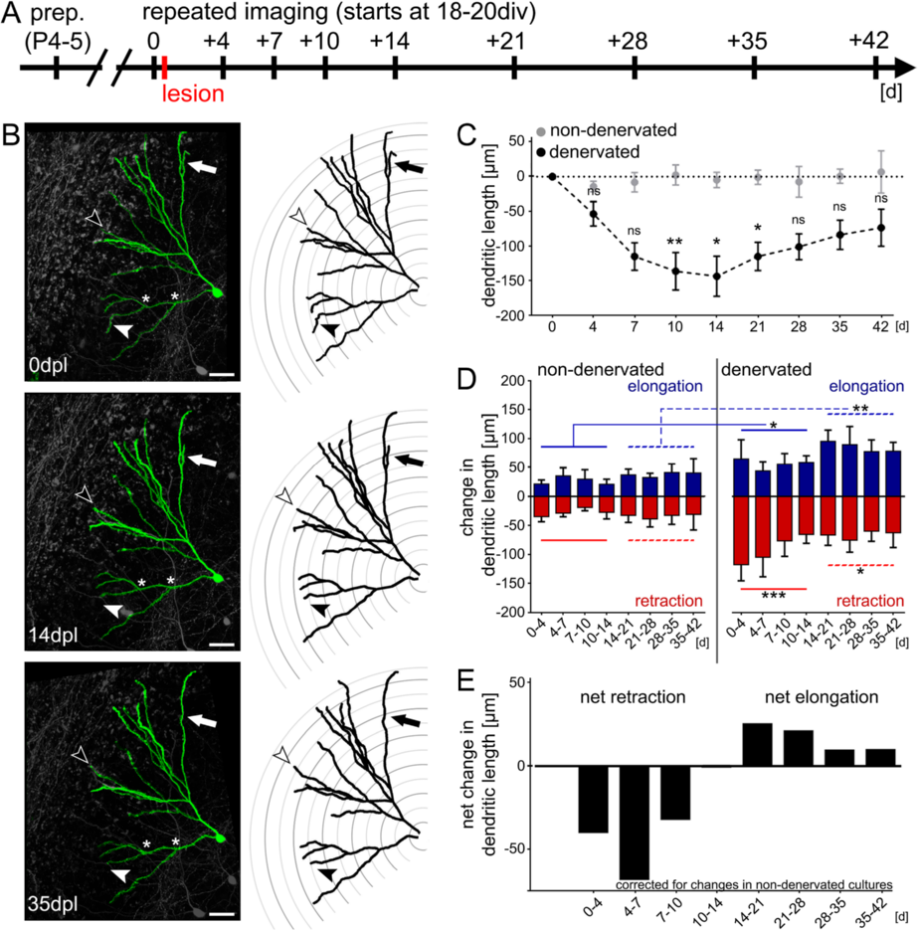
Denervation-induced dendritic remodeling. **a** Schematic of the experimental design. Slice cultures were prepared at postnatal day 4–5 and allowed to mature for 18–21 days in vitro (div). Repetitive imaging was performed at indicated points in time. Cultures were lesioned immediately after the first imaging session. **b** Example of a denervated dentate granule cell (2D-projected confocal image stack) and the corresponding reconstructions are shown for 0, 14 and 35 days post lesion (dpl). Note retraction (black arrowhead) and elongation (white arrowhead) of distal dendritic segments. The complete loss of a distal dendritic branch is indicated by the black arrow. Scale bar: 40 μm. **c** Entorhinal denervation in vitro leads to a reduction in the total dendritic length (TDL) of granule cells, which is followed by a gradual recovery in TDL. No significant change in mean TDL was seen in age- and time-matched non-denervated control cultures imaged in the same way (*n* = 6 non-denervated cultures vs. *n* = 8 denervated cultures; Kruskal-Wallis-test followed by Dunn’s post-hoc-test; *, *p* <0.05; **, *p* <0.01; ns, not significant). **d** Analysis of dendritic retraction and elongation reveals a higher degree of dendritic remodeling in denervated cultures. Both retraction and elongation are increased in the denervated group throughout the observation period (Wilcoxon-Mann–Whitney test with pooled data 0-14d and 14-42d; *, *p* <0.05; **, *p* <0.01; ***, *p* <0.001). **e** Net effects of dynamic changes in the dendritic tree demonstrate net retraction during the early phase and net elongation during the late phase after denervation. Data corrected for changes in non-denervated cultures

Live imaging of slice cultures was performed as previously described [21, 22]. Briefly, slice cultures on the filter inserts (Millipore, Germany) were transferred to a petri dish containing preheated (35 °C) imaging medium (NaCl 129 mM, KCl 4 mM, MgCl_2_ 1 mM, CaCl_2_ 2 mM, glucose 4.2 mM, HEPES 10 mM, Trolox 0.1 mM, streptomycin 0.1 mg/ml, penicillin 100 U/ml; pH 7.4; osmolarity adjusted with sucrose to match the osmolarity of the cultivation medium). Filter inserts were secured by a custom made titanium ring. The cultures were viewed with an upright Zeiss LSM Pascal confocal microscope. A 10x water immersion objective (0.3 NA, Zeiss, Germany) was used to visualize the culture at a low magnification to identify individual granule cells. Then a 40x water immersion objective (0.9 NA; Zeiss, Germany) was used to image the dendritic tree of a single granule cell. Up to 40 images were recorded per stack (512 × 512 pixel, 0.11 μm/pixel; z-steps: 3 μm). Per filter insert (containing up to six cultures) one identified granule cell was visualized to minimize dwell time during imaging procedure (<10 min per culture). The dendritic trees of individual GFP-expressing granule cells of denervated and non-denervated age- and time-matched control cultures were repeatedly imaged for up to 6 weeks, i.e., 42 days post lesion (dpl; Fig. 2a) using the same imaging procedure and settings at the microscope.

### Drugs

Denervated and non-denervated slice cultures were treated with FTY720 (1 μM; Selleck Chemicals, 162359560), VPC23019 ([28]; 1 μM; VPC23019, R- Phosphoric acid mono-ester; Tocris 4195) or S1P (1 μM, Biotrend, BS0186) by applying the respective drug (or vehicle-only) after the first imaging session (and immediately after the lesion; c.f., Fig. 2a) to the incubation medium and to the imaging solution. Drug- or vehicle-containing incubation medium was replaced three times per week. We did not observe any evidence for toxicity (blebbing, dendritic atrophy/retraction) in our time lapse imaging experiments of non-denervated cultures treated with either FTY720 or VPC23019. In these imaging experiments the control cells were stable and not a single imaged cell was lost under these conditions. Thus, we feel confident that our observations are not confounded by a toxic effect of these drugs on neural tissue.

### Reconstruction of the dendritic tree

**Fig. 3:**
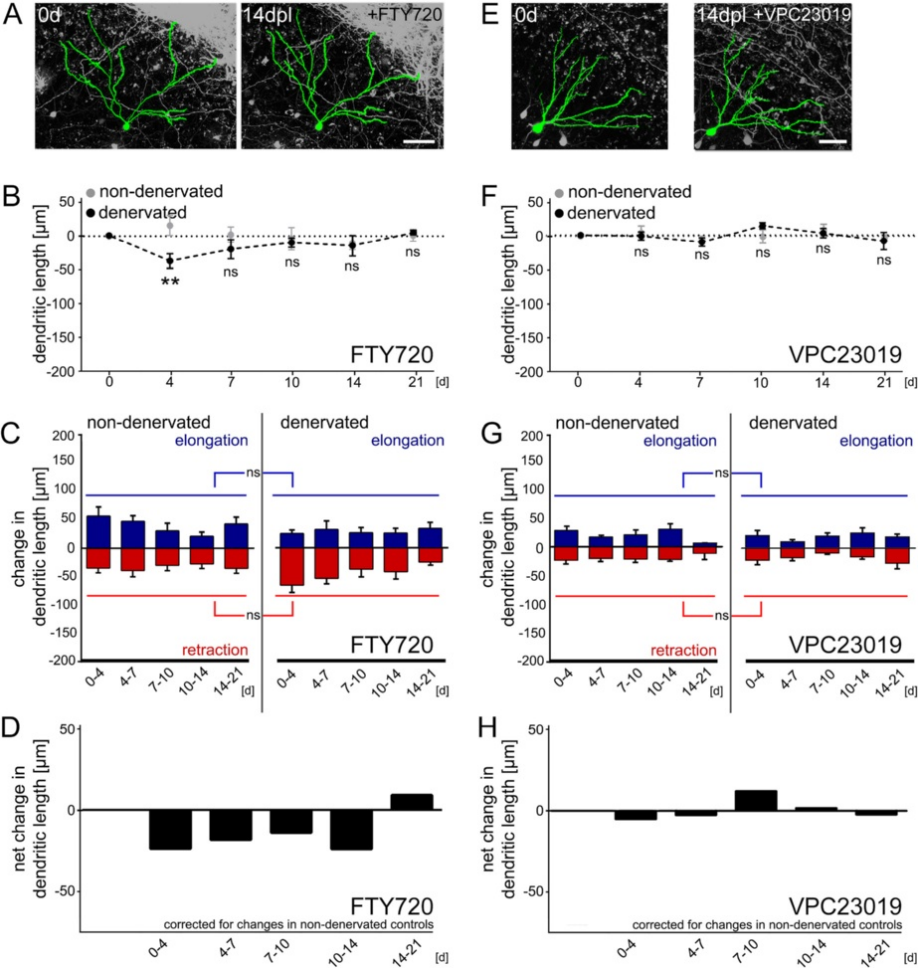
Sphingosine-1-phosphate (S1P) receptor inhibition prevents denervation-induced dendritc remodeling and stabilizes deafferented dendrites. **a**-**d** Application of FTY720 (1 μM) into the incubation medium immediately after entorhinal denervation in vitro **a**, **b** prevents the protracted reduction in total dendritic length (TDL; *n* = 9 neurons per group, one cell per culture; Kruskal-Wallis-test followed by Dunn’s post-hoc-test; **, *p* <0.01; ns, not significant). An initial retraction is observed, which is consistent with the agonist–antagonist properties of FTY720, initially leading to the activation of S1P receptors and their subsequent internalization. **c**, **d** FTY720 prevents the denervation-induced destabilization of dendrites (Wilcoxon-Mann–Whitney test with pooled data 0 - 21d; ns, not significant), while having no apparent effect in non-denervated cultures (statistically compared against vehicle-treated cultures, data taken from Fig. 2; Kruskal-Wallis-test followed by Dunn’s post-hoc-test; not significant, not shown). **e**-**h** Similar results were obtained in a different set of experiments, in which the S1P-receptor 1 and 3 inhibitor VPC23019 (1 μM) was used **e**: no change in (F) TDL (*n* = 7 neurons per group, one cell per culture; Kruskal-Wallis-test followed by Dunn’s post-hoc-test; ns, not significant) and **g** dendritic elongation and retraction (Wilcoxon-Mann–Whitney test with pooled data 0 - 21d; not significant) following denervation. VPC23019 had no apparent effect on dendrites of granule cells in non-denervated cultures (statistically compared against vehicle-treated cultures, data taken from Fig. 2; Kruskal-Wallis-test followed by Dunn’s post-hoc-test; not significant, not shown) and **h** clearly prevented the net retraction following entorhinal denervation in vitro. Scale bars in A and E: 50 μm

The dendritic tree of imaged single dentate granule cells was manually reconstructed in confocal image stacks using SpineLab [27]. Total dendritic length (TDL) was calculated as the sum of length of each individual reconstructed dendritic segment of an identified neuron. In addition, changes in the length of identified dendritic segments of a neuron were monitored over time, i.e., between each consecutive point in time to determine elongation and retraction of neurons (Fig. 2, 3 and 5).

### Mass spectrometry

**Fig. 4:**
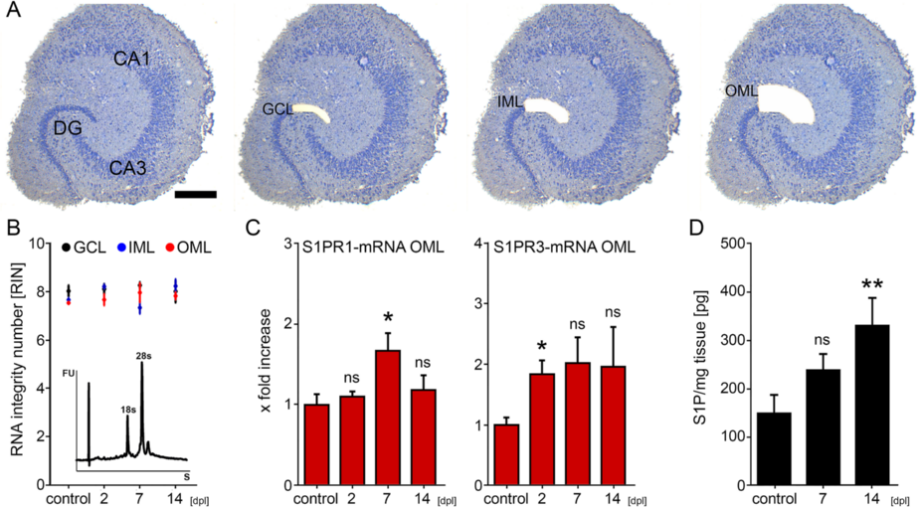
Change in Sphingosine-1-phosphate (S1P) and S1P receptor (S1PR) 1 and 3 mRNA levels following entorhinal denervation in vitro. **a** Laser microdissection (LMD) was employed to collect tissue from the granule cell layer (GCL) the inner molecular layer (IML) and the outer molecular layer (OML) of denervated cultures (at 2, 7 and 14 days post lesion; dpl) and non-denervated cultures (control; age- and time-matched to denervated cultures). Scale bar: 50 μm. **b**, **c** LMD/qPCR results of non-denervated and denervated cultures. **b** RNA integrity numbers (RIN) for the acquired samples. S1PR1- and S1P3-mRNA levels were assessed in the isolated tissue using qPCR. **c** An increase in S1PR1-mRNA was observed in the OML at 7 dpl. Similarly, increased S1PR3-mRNA levels were observed in the OML (at 2 dpl). S1PR1- and S1PR3-mRNA levels in the IML and GCL were not significantly changed (data not shown; *n* = 4–9 cultures per group; Kruskal-Wallis test followed by Dunn’s multiple comparisons test: *, *p* <0.01; ns, not significant; age- and time-matched non-denervated controls were not significantly different and therefore pooled). **d** Mass spectrometry disclosed a gradual increase in S1P levels in denervated hippocampal tissue as compared to samples taken from age- and time-matched non-denervated cultures (control; *n* = 9–11 independent experiments per group; Kruskal-Wallis test followed by Dunn’s multiple comparisons test: **, *p* <0.01; ns not significant; age- and time-matched non-denervated controls were not significantly different and therefore pooled)

S1P concentrations were determined in slice culture tissue as described in [29]. After addition of internal standards (C17-Sphingosine-1-phosphate) and liquid extraction using chloroform: MeOH:HCl (83:15:2, v/v/v), HPLC separation was done under gradient conditions using a Luna C18-column (150 cm × 2 mm, Phenomenex, Germany). MS/MS analyses were performed on a API4000 triple quadrupole mass spectrometer with a Turbo V source (AB Sciex, Germany) operated in positive ionization mode, as described in detail previously (Fig. 4d; [29]). Concentrations of the calibration standards, quality controls and samples were evaluated by Analyst software version 1.5 (AB Sciex, Germany) using a standard curve. The coefficient of correlation for all measured sequences was at least 0.99. Variations in accuracy and intra-day and inter-day precision (*n* = 6 for each concentration, respectively) were <15 % over the range of calibration.

### Laser capture microdissection (LMD) of re-sliced cultures

Slice cultures were washed with phosphate buffered saline (PBS; 0.1 M, pH 7.4), shock frozen at −80 °C in tissue freezing medium (Leica Microsystems, Germany), re-sliced into 10 μm thick slices on a cryostat (Leica CM 3050 S) and mounted on PET foil metal frames (Leica, Germany) as described previously [20, 26]. Re-sliced cultures were fixed in ice-cold acetone for 1 min and incubated with 0.1 % toluidine blue (Merck, Germany) at room temperature for 1 min, before rinsing in ultrapure water (DNase/RNase free, Invitrogen, USA) and 70 % ethanol. PET foil metal frames were mounted on a Leica DM 6000B LMD system (Leica Microsystems, Germany) with the section facing downward. After adjusting intensity, aperture, and cutting velocity, the pulsed ultraviolet laser beam was carefully directed along the borders of the respective hippocampal layers of interest using a 20x objective lens (Leica Laser Microdissection, Software Version 7.4.1.4853). Tissue from the outer and inner molecular layer (OML, IML) and the granule cell layer (GCL) of the suprapyramidal blade of the dentate gyrus were collected (Fig. 4a). Microdissected tissue was transferred by gravity into microcentrifuge tube caps placed underneath the sections, filled with 50 μl guanidine isothiocyanate (GITC)-containing buffer (RLT Buffer, RNeasy Mini Kit, Qiagen) with 1 % ß-mercaptoethanol (AppliChem GmbH, Germany). Successful tissue collection was verified by visually inspecting the content of the tube caps. All samples were frozen and stored at −80 °C.

### Isolating RNA and qPCR

RNA was isolated using the RNeasy® MicroPlus Kit (Qiagen, Germany). RNA integrity number (RIN; Fig. 4b) was determined using the Agilent 2100 Bioanalyzer system and Agilent RNA 6000 Pico Kit (Agilent Technologies, Germany). Purified RNA was transcribed into cDNA with the High Capacity cDNA Reverse Transcription Kit (Applied Biosystems, USA). All kits and assays were used according to the manufacturer’s instructions. The cDNA was amplified using the TaqMan®PreAmp Master Mix Kit (Applied Biosystems, USA) using 5 μl PreAmp Master Mix (Applied Biosystems, USA) + 2.5 μl cDNA + 2.5 μl Assay Mix [TaqMan Gene Expression™-Assay; GAPDH: 4352932E; sphingosine-1-receptor 1 (S1PR1) Mm00514644_m1eas; sphingosine-1-receptor 3 (S1PR1R3) Mm02620181_s1 from Applied Biosystems, USA] with a standard amplification protocol (14 cycles: 95 °C for 15 s; 60 °C for 4 min). Amplified cDNAs were diluted 1:20 in ultrapure water and subjected to qPCR (Fig. 4c; StepOnePlus, Applied Biosystems, USA) using a standard amplification program (1 cycle of 50 °C for 2 min, 1 cycle of 95 °C for 10 min, 40 cycles of 95 °C for 15 s and 60 °C for 60 s; cut off at 30 cycles; average C_T_-value was: 20.0 ± 0.9 cycles).

### Quantification and statistics

To minimize error, segments of cultures with entorhinal lesions were compared to segments of age- and time-matched cultures without lesions, which were imaged the same number of times using the same imaging protocol. Every analysis was performed with the person analyzing dendritic morphologies blind to experimental condition.

qPCR-data were analyzed as described by Pfaffl [30]. GAPDH served as reference gene in this analysis. The qPCR assay efficiency was calculated with the StepOnePlus software (Applied Biosystems, USA) based on a dilution series of 5 samples for each assay. Data of non-denervated control cultures (age- and time-matched to denervated cultures) were pooled.

Statistical comparisons were made using non-parametric Wilcoxon-Mann–Whitney test or the Kruskal-Wallis-test followed by Dunn’s post-hoc-test, which takes multiple comparisons into consideration. *P*-values of less than 0.05 were considered significant. All values represent means ± standard error of the mean (sem). In the figures * denotes *p* <0.05, ** *p* <0.01 and *** *p* <0.001; not significant differences are indicated with ‘ns’.

### Digital Illustrations

Confocal image stacks were exported as 2D-projections from the Zeiss LSM image browser and stored as TIF files. Figures were prepared using Photoshop graphics software (Adobe, USA). Image brightness and contrast were adjusted.

## Results

### Denervation induces a loss in total dendritic length

Time-lapse microscopy of organotypic slice cultures prepared from Thy1-GFP mice [23] was used to determine the dynamics of denervation-induced dendritic remodeling. Single denervated GFP-expressing granule cells and single age- and time-matched non-denervated GFP-expressing granule cells were repeatedly imaged over time (Fig. 2a, b). Changes in total dendritic length (TDL) were determined in the two groups (Fig. 2c). Entorhinal denervation in vitro caused a decrease in TDL during the first 2 weeks after lesion, which was followed by a gradual recovery in TDL at later time points (>14 days post lesion, dpl). Control cultures did not show any significant change in TDL over time. These data are consistent with previous studies demonstrating reduction in dendritic length followed by partial recovery in TDL upon entorhinal denervation in vivo [31, 32]. They confirmed the validity of our in vitro model to study the cellular and molecular mechanisms of denervation-induced dendritic remodeling.

### Denervation affects the dynamics of dendrites and causes loss of dendritic segments

In order to assess the dynamics of the lesion-induced dendritic reorganization process, we compared neuronal reconstructions of granule cells at consecutive time points (Fig. 2d). This made it possible to assess elongation and retraction of individual dendritic segments separately. In control cultures changes of distal dendritic segments were detected under baseline conditions (within a range of ± 30–40 μm between imaging sessions). Approximately 5–7 % of the TDL was found to be dynamically remodeled between consecutive points in time. These alterations did not directly affect TDL since elongation and retraction events canceled out. Dendritic arborization was also unchanged, since neither loss of existing nor formation of new dendritic segments was seen. We concluded that under control conditions dendritic dynamics are in a homeostatic steady-state and TDL remains constant.

In the denervated group time-lapse imaging revealed profound changes in dendritic dynamics and arborization (Fig. 2d). In some cases individual segments disappeared without reappearing during the recovery phase (see arrow in Fig. 2b). Formation of new dendrites was not observed. Much to our surprise dendritic retraction and elongation were elevated during the early and the late phase after denervation. These results suggested that atrophic and compensatory dendritic changes occur in parallel and are both detectable even at a late stage after the lesion, i.e., when TDL recovers. During the early phase (0–14 dpl) retraction exceeded elongation, while at a later stage (>14 dpl) elongation surpassed retraction (Fig. 2d, e). These changes in dendritic dynamics were sufficient to explain the time course of granule cell TDL following entorhinal denervation in vitro.

### Inhibition of S1P-receptor signaling maintains granule cell dendrites after denervation

To test for the role of FTY720 and S1PR signaling in denervation-induced dendritic remodeling, we repeated deafferentation experiments in a new set of cultures which were treated with FTY720 (1 μM) immediately after the lesion. Non-denervated FTY720-treated cultures served as controls (Fig. 3a-d). These cultures were imaged in the same way as the denervated cultures. Strikingly, FTY720 treatment prevented the denervation-induced loss in TDL. Only at 4 dpl a significant reduction in TDL was briefly observed. Analysis of dendritic elongation and retraction revealed that FTY720 acts by stabilizing granule cell dendrites, i.e., by preventing the denervation-induced increase in dendritic remodeling. Of note, the dendritic arbor was also maintained since dendritic segments were neither lost nor newly formed after deafferentation.

To confirm and extend these results, another set of control and denervated cultures was treated with VPC23019 (1 μM), which is a competitive S1PR1/3 inhibitor (Fig. 3e-h [28]). In these experiments no changes in TDL were observed over time, both in VPC23019-treated denervated and non-denervated cultures. Very similar to FTY720, VPC23019 also prevented the denervation-induced destabilization of dendrites. Statistical comparison of these results with data obtained in cultures that were not pharmacologically treated (c.f., Fig. 2) confirmed that neither FTY720 nor VPC23019 affect TDL and dendritic dynamics under non-denervated control conditions, while preventing the denervation-induced dendritic changes (Kruskal-Wallis-test followed by Dunn’s post-hoc-test).

Taken together, these findings suggested that inhibition of S1PR signaling prevents denervation-induced dendritic atrophy by stabilizing the dendrites of partially deafferented neurons. Since the peripheral immune system is absent in slice culture preparations, these results indicated that FTY720 could act directly on neural tissue [33–35].

### Upregulation of S1P-receptor 1 and 3 mRNA in the denervated outer molecular layer following entorhinal denervation in vitro

Because both FTY720 and VPC23019 act via S1PRs, we wondered whether these receptors are expressed and regulated in the dentate gyrus following denervation (Fig. 4). We focused on S1PR1 and S1PR3 in these experiments, since earlier work indicated that FTY720 acts predominantly via these receptors [16, 36, 37]. Laser capture microdissection (LMD) was used to harvest tissue from the denervated OML, the non-denervated IML and the GCL in control and denervated cultures at 2, 7 and 14 dpl (Fig. 4a). RNA integrity numbers (RIN) of harvested tissue was excellent (Fig. 4b). Changes in mRNA levels were determined in laser microdissected material by qPCR. A significant increase in S1PR1- and S1PR3-mRNA levels was detected in the denervated OML (Fig. 4c), while no significant changes were observed in the IML and GCL (data not shown). S1PR3-mRNA was increased at 2 dpl, while S1PR1-mRNA showed a later increase at 7 dpl (Fig. 4c).

### S1P levels are upregulated after denervation

We next tested whether entorhinal denervation leads to an increase in S1P levels in hippocampal tissue (Fig. 4d). Indeed, mass spectrometry disclosed a gradual increase of S1P levels in denervated cultures, reaching the level of significance by 14 dpl in comparison to non-denervated controls. Together with our LMD/qPCR data these results demonstrated an upregulation of both the ligand as well as the receptors in the dentate gyrus following denervation.

### S1P-treatment is not sufficient to induce dendritic atrophy in control cultures

**Fig. 5:**
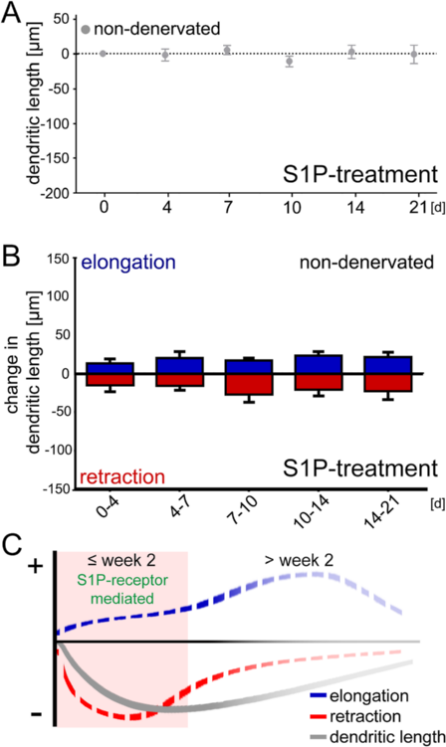
Sphingosine-1-phosphate (S1P) treatment does not affect the dynamics of granule cell dendrites in non-denervated control cultures. **a**, **b** Application of exogenous S1P (1 μM) into the incubation medium did not reduce the total dendritic length (TDL) of dentate granule cells in non-denervated cultures **a** and did not cause dendritic destabilization, i.e., changes in dendritic elongation and retraction **b** (*n* = 6 neurons per group; one cell per culture; statistically compared against untreated controls, pooled, taken from Fig. 2; Kruskal-Wallis-test followed by Dunn’s post-hoc-test; ns, not significant). **c** Schematic illustration of the *stability model* of denervation-induced dendritic remodeling. The results of the present study demonstrate that partial deafferentation leads to profound changes in dendritic stability. Both, elongation and retraction of dendritic segments are increased after entorhinal denervation. During the early phase, retraction exceeds elongation, which results in a reduction of TDL. At a later stage elongation surpasses retraction and TDL recovers. Our data suggest that S1P-receptor signaling prevents these denervation-induced changes in dendritic stability and, thus, changes in TDL

To determine whether high levels of S1P are sufficient to destabilize dendrites and to induce dendritic atrophy yet another set of non-denervated cultures was treated with S1P (1 μM, i.e., 379 ng/ml). Again, individual dentate granule cells were repeatedly imaged over time. In these experiments neither a reduction in TDL (Fig. 5a), nor an increase in dendritic elongation or retraction (Fig. 5b) were observed. We conclude that high S1P-levels in the culture medium are not sufficient to trigger the destabilization of granule cell dendrites *per se.*

## Discussion

Neurological diseases associated with neuronal cell death show primary damage at the lesion site and widespread secondary damage in connected brain regions. Secondary damage, mainly caused by the loss of innervating axons originating from neurons at the primary lesion site, severely disrupts otherwise unaffected and healthy brain areas and perturbs network function. Of note, secondary damage is largely independent of the underlying cause of the disease and almost invariably accompanied by neuronal atrophy [6, 38]. Even though secondary brain damage has now been recognized as a major factor contributing to neurological diseases, it has not been targeted for therapeutic intervention. We regard it as one of the major findings of our study that a clinically used immune-modulating drug, i.e., FTY720, is able to act directly on neural tissue and prevents transneuronal denervation-induced dendrite loss. This effect is mediated by inhibition of neural S1P signaling. Considering that an increase in S1P-levels and S1P-receptor mRNAs is also detected after deafferentation, we propose that clinically used S1PR-modulators may act directly on denervated neuronal networks irrespective of the underlying cause of the disease.

### Denervation affects dendritic stability and results in the rarefication of the dendritic arbor

Transneuronal degeneration of neurons after denervation has been well-described by several authors in different species and brain regions using in vivo lesions and perfusion-fixed tissue [3, 4]. We recently revisited this phenomenon and assessed changes in granule cell dendrites following entorhinal denervation in Thy1-GFP mice in vivo [32]. Using the same approach as in these earlier studies, we reported a protracted loss of dendrites, i.e., the rarefication of the dendritic arbor, which was followed by partial recovery of TDL at a later stage after denervation. Of note, in all of these studies - including our own - these changes were interpreted as the result of an initial degenerative and atrophic process followed by a partial regrowth of dendrites at later time points.

By using organotypic slice cultures, in vitro lesions and time-lapse imaging, we developed an in vitro system, which can be used to image the same denervated neuron over time and which makes it possible to study transneuronal changes dynamically [21, 22]. This approach revealed a constant remodeling of distal granule cell dendrites, similar to what has been reported for pyramidal cell dendrites in vivo using cranial windows [39]. In control cultures the retraction and elongation of dendritic segments appeared to be in a well-tuned, i.e., homeostatic, equilibrium. Thus, TDL remained stable over long observation periods. As predicted, denervation destabilized granule cell dendrites and increased the retraction of dendrites. In some cases entire segments were lost, which died back to the branch point and disappeared. However, increased dendritic retraction was also observed during the recovery phase of TDL (>14 days post lesion). Moreover and quite unexpectedly, dendritic elongation was also increased immediately after the lesion. During the phase of TDL loss (first 2 weeks), retraction exceeded reactive elongation, whereas during the phase of TDL recovery (weeks 3–6) elongation exceeded retraction. In sum, denervation does not result in the expected sequential change of dendrite loss followed by a period of dendrite regrowth. Rather, denervation causes a profound and long-lasting destabilization of granule cell dendrites, which involves increases in both elongation and retraction. Changes in TDL which accompany denervation are the result of an altered balance between these two phenomena.

### FTY720 treatment results in the stabilization of denervated dendrites

Based on reports that S1P-receptor signaling is involved in neuronal plasticity (e.g., [34, 40, 41]), regulation of neurite remodeling (e.g., [40, 42]), and neuroprotection (e.g., [33, 43, 44]) and also because of the relevance of FTY720 in the treatment of MS, we wondered whether FTY720 could have an influence on denervation-induced dendritic changes. Indeed, FTY720 prevented the denervation-induced reduction in TDL. Furthermore, dynamic imaging revealed that FTY720 stabilizes denervated dendrites and thus prevents the denervation-induced change in the balance of dendritic retraction and elongation. This *"neuro-stabilization"* seems to be a direct effect of FTY720 on neural tissue, since the peripheral immune system, which is regarded as the main target of FTY720, is missing in organotypic slice culture preparations. To control for off-target effects of FTY720 (and its agonist–antagonist properties; [16–18]), we repeated these experiments with the S1PR1/3-inhibitor VPC23019 and obtained very similar results. Hence, together with our LMD-qPCR and mass spectrometry data - which disclose an increase in both the ligand and the receptor - we are confident to conclude that S1P signaling pathways are involved in mediating denervation-induced plasticity.

Which cell biological processes could be relevant? At present little is known about the cellular and molecular mechanisms regulating denervation-induced dendritic retraction and elongation in adult neurons. Changes in neuronal cytoskeleton, e.g., changes in microtubule dynamics, are necessary for the formation of dendritic arbors during development and similar mechanisms are likely to play a role. Whether these cellular changes are brought about by external signals deriving from degenerating axon terminals or activated glial cells or are a response of the neuron to changes in its afferent activity is currently unclear. Likewise, glial cells [45–47], neurotrophic factors [33, 42], histone acetylation [48], and other aspects of injury-induced neuroinflammation [6, 38] could play a role. Notably, recent work has suggested that FTY720 may prevent p75NTR up-regulation and astrocyte-mediated inflammation [49]. Considering that these signaling pathways are known to affect dendritic morphology [50, 51] and our own recent work on TNF in denervation-induced plasticity [26, 52], it is possible that FTY720 could act through p75NTR in our experimental setting. Regardless of these considerations, which show that we urgently need a more thorough molecular understanding of disease-related neuronal reorganization processes, our data clearly show that FTY720 prevents structural changes of denervated granule cells and stabilizes their dendritic arbor in the absence of their specific input.

### The S1P signaling pathway is permissive for dendritic remodeling

Since FTY720 prevented denervation-induced dendritic changes of granule cells, we hypothesized that S1PRsignaling is involved in this process. Therefore, we investigated changes in S1P after denervation and found, by using mass spectrometry, that S1P is present in our preparations and that levels of S1P increase following entorhinal denervation. Since VPC23019 had similar effects as FTY720, we focused on S1PR1 and S1PR3 as the most likely S1P-receptors to be involved. Indeed, mRNAs of both receptors were found to be present and upregulated in the denervated outer molecular layer of the dentate gyrus [53]. Together, these data strongly implicate S1P and S1P signaling via S1PR1 and S1PR3 in denervation-induced dendritic remodeling.

Interestingly, neither addition of S1P (at ~10^3^x higher concentration compared to the measured endogenous S1P-levels in denervated cultures) nor the addition of either FTY720 or VPC23019 to the culture medium had effects on granule cell dendrites and their dynamics in non-denervated control cultures. These experiments demonstrate that S1P signaling is neither sufficient nor instructive for the induction of dendritic changes, nor is it necessary for the maintenance of non-denervated dendrites (c.f., [54]). Postlesional changes of S1PR or their signaling pathways might be required for granule cells to become sensitive to S1P. Under such conditions S1P could influence the dynamics of dendrites and could regulate dendritic remodeling. From a therapeutic point-of-view such an *“effect-dependence”* on denervation conditions is of considerable benefit, since treatment with FTY720 will not alter the dynamics of dendrites of non-denervated neurons in other brain areas.

### Clinical implications

FTY720 is an orally active drug used in clinical medicine for the treatment of MS [8–10]. It is believed to act primarily on circulating lymphocytes and lymphnodes, i.e., immune cells outside the CNS [55] (for review see [16, 56]). Consistent with work of other groups [33–35] our data disclose that FTY720 can also interfere with S1P signaling in neural tissue, and can prevent a loss of dendrites upon deafferentation. Although the functional consequences of dendritic atrophy and dendritic reorganization on a denervated neuronal network are still unclear and some authors have even suggested homeostatic functions [57], it is attractive to speculate that the ability of FTY720 to stabilize denervated dendrites is one of the reasons why this drug has a beneficial effect for MS patients. For example, during periods of neuroinflammation FTY720 could maintain partially denervated dendrites until surviving or recovering axons reinnervate the dendritic tree. Thus, FTY720 could prevent a loss of dendritic complexity as seen in long-term denervated neurons [31, 32], which may limit a neuron’s ability to process and integrate information from different inputs.

## Conclusion

Entorhinal denervation causes profound and long-lasting destabilization of granule cell dendrites. Changes in dendritic length are the result of an altered balance between elongation and retraction of dendrites: During an early phase after denervation dendritic retraction exceeds elongation, followed by a later stage during which elongation surpasses retraction. Inhibition of S1PR signaling prevents dendritic destabilization and denervation-induced dendrite loss. Whether or not FTY720 or other drugs acting via S1PR pathways could minimize secondary damage to neurons in MS or in other neurological diseases now needs to be investigated using appropriate in vivo models.
